# Redefining and Validating Digital Biomarkers as Fluid, Dynamic Multi-Dimensional Digital Signal Patterns

**DOI:** 10.3389/fdgth.2021.751629

**Published:** 2022-01-13

**Authors:** Rhoda Au, Vijaya B. Kolachalama, Ioannis C. Paschalidis

**Affiliations:** ^1^Department of Anatomy and Neurobiology, Neurology and Framingham Heart Study, Boston University School of Medicine, Boston, MA, United States; ^2^Department of Epidemiology, Boston University School of Public Health, Boston, MA, United States; ^3^Boston University Alzheimer's Disease Center, Boston, MA, United States; ^4^Department of Medicine, Boston University School of Medicine, Boston, MA, United States; ^5^Faculty of Computing and Data Sciences, Boston University, Boston, MA, United States; ^6^Department of Electrical and Computer Engineering, Division of Systems Engineering, and Department of Biomedical Engineering, Boston University, Boston, MA, United States

**Keywords:** digital, technology, biomarkers, digital biomarkers, fluidic dynamic digital patterns, new regulatory standards

## Abstract

“Digital biomarker” is a term broadly and indiscriminately applied and often limited in its conceptualization to mimic well-established biomarkers as defined and approved by regulatory agencies such as the United States Food and Drug Administration (FDA). There is a practical urgency to revisit the definition of a digital biomarker and expand it beyond current methods of identification and validation. Restricting the promise of digital technologies within the realm of currently defined biomarkers creates a missed opportunity. A whole new field of prognostic and early diagnostic digital biomarkers driven by data science and artificial intelligence can break the current cycle of high healthcare costs and low health quality that is being driven by today's chronic disease detection and treatment approaches. This new class of digital biomarkers will be dynamic and require developing new FDA approval pathways and next-generation gold standards.

The term “digital” is associated with a task that typically uses sensors and computational tools, generally across multiple layers of hardware and software. The plethora of physiological and behavioral data acquired via various digital streams allows for the pursuit of “digital biomarkers.” Most common forms of collecting digital data continuously include web-based applications, smartphones, wearables, and even via implantable or digestible devices. Increasing interest in digital data collection is a reflection of the latest technological advances, and this has raised the hope of creating frameworks for better and healthy living, as well as improved outcomes. More often than not, any characterization of health-related behaviors or disease-related symptoms that is digitally collected is indiscriminately being labeled as a “digital biomarker.” As such, there is a common misconception about the definition of a digital biomarker as an online extension of a traditional biomarker. This problem is further exacerbated by the conflating of the “digital biomarkers” to the same identification and validation pathways of well-established and United States Food and Drug Administration (FDA) approved preclinical and diagnostic biomarkers [e.g., pre-cancerous cells, amyloid-ß in the blood, cerebrospinal fluid (CSF) or through Positron Emission Tomography (PET), cardiac enzymes of heart failure, etc.] (1). We need to recognize the differences between amyloid-ß measured from a PET scan at a single timepoint compared to physical activity or sleep data that is collected continuously and passively using a mobile device for a period of weeks, months, or even years. While the former (i.e., PET-based amyloid-ß) is an illustration of a traditional FDA-approved biomarker (2), the dynamic measure of physical activity and sleep health obtained from a time series analysis of yearlong data may not necessarily fit within the framework of a traditional biomarker. Further, even standard characterization of physical activity and sleep differentiate the two rather than combining them into a metric that more accurately detects both movement and sleep behaviors across a 24-h cycle. Sleep disordered behaviors can lead to greater physical activity at night and less during the day, or sleep can also occur both regularly or sporadically during conventional wakefulness day time hours.

The FDA follows the Biomarkers, EndpointS, and other Tools (BEST) glossary to define a biomarker (3). It is measured as an indicator of normal biological or pathogenic process, or a response to an exposure or intervention. The FDA notes that any molecular, histologic, radiographic, or physiologic characteristics are types of biomarkers, whereas an assessment of how an individual feels, functions, or survives is not considered as a biomarker. If we stay true to this definition, then it is not trivial to identify and validate a 24-h continuous measure of physical activity and sleep that can serve as a biomarker. The primary reason is because passively and digitally collected data streams are not necessarily static stamps of health but a conglomeration of sensor-derived numerics that span across the daily living of individuals in various environments in a 24-h wake-sleep cycle. If we simplify all the variability from this complex physical activity-sleep calculus into a single measure to fit the classic definition of a biomarker, then we are doubtlessly diminishing the overall value that can be derived from the stream of information that is collected from various sensors. One might argue that such simplication of complex activities to a scalar metric can produce actionable insights but the richness of information is effectively lost in that process. Thus, there is an urgent need to work with the FDA to revisit the term “digital biomarker” insomuch to create a unique pathway toward a safe and sustained development of novel biomarkers for health and disease using digital data streams. Fortunately, the FDA does not have to work on the development of these definitions in isolation. Some steps toward making a distinction between approval processes for traditional vs. digital biomarkers have been taken by the European Union (EU) medicines agency (4).

## Recommendations

There are several recommended steps in how to do so:

1. Review current definitions of biomarkers and clearly define where the term “digital biomarker” applies and does not.

- For example, a static blood-based measurement of C-reactive protein is a traditional biomarker to detect inflammation in the body. On the other hand, a voice recording of an individual collected continuously or intermittently over various conversations and even during a medical visit can be processed to derive a dynamic, quantitative signal of their speech pattern, which in turn can serve as a marker of the individual's cognitive status (5). Similaly, the digital image of a drawing may be used for cognitive assessment (6). This measure can fall within the category of a digital biomarker.
- In essence, while static measures of patient-level characteristics fall into the realm of traditional biomarkers, information that is multidimensional, temporally varying, and multimodal can be synthesized in the form of digital biomarkers. Note that this definition is not encompassing all forms of dynamic measures but only providing a baseline definition to what can be perceived as novel digital biomarkers.

2. Refine existing categories of digital biomarkers that includes distinguishing classes of digital biomarkers. A surrogate digital biomarker would correlate highly with a traditional biological biomarker, while a more novel type of digital biomarker would increasingly be less correlated directly to the biological one (e.g., proximal digital biomarker).

- For example, a new method for measuring a biological biomarker through a wearable device (e.g., a wearabale glucose monitor) can be considered as being in the first class because it reliably correlates with the biological one, whereas recording of behavioral or activity-based data that can reliably detect disease independent of the biological indicator belongs to the second class of “novel” digital biomarkers. This distinction does not necessarily imply that a new method for measuring a biological biomarker has no added value; for instance, it may enable continuous measurements. Still, the measurements are of an “established” biological biomarker. It is necessary to provide expanded clarity on what types of digital characterization of symptom/disease would fall under the definition of a digital biomarker and which would not.

3. Map out the FDA approval process one would have to follow based on the current regulatory pathways for each digital biomarker category and identify critical gaps that are barriers to approval for different conceptualization of digital biomarkers, particularly those that will not rely on validation through biological biomarkers, such as is described in point #2 above.
4. Characterize the current digital biomarker pathway to pre-market submissions that are made to the FDA and delineate recommended additions that would create new pre-market submission pathways for the new conceptualizations of digital biomarkers beyond those that exist today.
5. Identify FDA-approved and FDA-cleared digital devices that are currently covered by Medicare and Medicaid and those that overlap or are independently covered by private insurance to help accelerate the push of available digital tools into the clinical care setting. The path to widespread acceptance of digital biomarkers is necessarily dependent on much greater adoption rates of digital technology and more generally as a matter of clinical practice. Thus, identifying and promoting use of existing technologies, whose costs are reimbursed through public or private health insurance, will help bring about market readiness for more novel technology-driven applications.

In considering how to unleash the full potential of technology generally, and digital biomarkers in particular, it is important to embrace the complexity of chronic diseases (7). Chronic diseases comprise 86% of healthcare services and costs in the U.S. and they are one of the primary reasons why high healthcare costs persist (8). Medical practice is still largely centered on waiting till disease symptom severity triggers a treatment intervention and because most conditions are chronic, that means continuous treatment till end of life. Increased lifespans are further driving up costs of chronic disease care (9). Technology provides an opportunity to break this cycle of high healthcare costs and low health quality. Since most chronic diseases are insidious in onset, there is an opportunity to develop a whole new field of prognostic digital biomarkers that are identified so early that current measurement standards would deem them within normal levels. This will enable early interventions and will usher a new class of therapeutics that have the potential to go beyond managing a chronic disease, including preventing it or delaying its onset.

This conceptualization of a new class of digital biomarkers will necessarily involve developing a new FDA approval pathway and the establishment of next generation gold standards that are untethered to current biomarker validation precedent. Methods to identify, validate, and eventually approve these digital biomarkers will be centrally driven by data science and artificial intelligence approaches, a field that is rapidly evolving and has demonstrated important successes, particularly in image classification, natural language processing, and speech recognition (10). Key to facilitating application of advanced analytics will be robust data accessibility to data scientists worldwide and equitable credit to those who make their data available. In this forward-thinking vision, digital data streams will generate dynamic signal patterns. Each pattern will be distinct at any one point of time, but aggregated together across time will consist of a sequence of unique multi-dimensional digital profiles. Together they will comprise a “digital biomarker trajectory” one that is never replicated in its exact composition, but nonetheless is highly predictive of the target assessment (11).

As an example, take the challenge of early detection of memory impairment that may signal the beginning of the long neurodegenerative process of Alzheimer's disease (AD). Now understood as a life course disease, AD risk is imparted through a mix of factors; some of which are modifiable and represent AD prevention opportunity (12). A digital memory biomarker in 1 month could consist of a different digital signal mix such as increased repetitive steps to a single location, a decrease in the diversity of locations visited, and digital voice patterns that indicate significant word finding problems in a social setting. The second month may include many steps throughout the house (e.g., indicator of searching for a misplaced item), digital voice patterns of narrower range of word choice in the home environment, and an instance of fast paced steps to the kitchen near the area of the stove. Each month's mix of signals will be varied and in combination unique, but together present a dynamically evolving pattern of behaviors that are reliably representative of a memory impairment (13). This is an oversimplified example of what a new world of digital biomarkers might look like. The call to action is to urge the science and technology community in lock step with the FDA, to begin the work of carving this digital biomarker pathway today.

## Data Availability Statement

The original contributions presented in the study are included in the article/supplementary material, further inquiries can be directed to the corresponding author/s.

## Author Contributions

RA conceptualized and wrote the initial draft. VK and IP provided critical review and edits. All authors contributed to the article and approved the submitted version.

## Funding

This project was supported in part by the National Center for Advancing Translational Sciences, National Institutes of Health, through BU-CTSI Grant (1UL1TR001430), a Hariri Research Award from the Hariri Institute for Computing and Computational Science and Engineering and Grant (GM135930) at Boston University and National Science Foundation Grants (DMS-1664644 and IIS-1914792), and National Institute on Aging Grants (AG062109, AG072654, AG013846, and AG016976). Additional support was provided by Boston University's Affinity Research Collaboratives Program, Alzheimer's Drug Discovery Foundation (201902-2017835) and Gates Ventures.

## Conflict of Interest

The authors declare that the research was conducted in the absence of any commercial or financial relationships that could be construed as a potential conflict of interest.

## Publisher's Note

All claims expressed in this article are solely those of the authors and do not necessarily represent those of their affiliated organizations, or those of the publisher, the editors and the reviewers. Any product that may be evaluated in this article, or claim that may be made by its manufacturer, is not guaranteed or endorsed by the publisher.
